# Translation and validation for Persian version of Abbreviated Moral Injury Outcome Scale (AMIOS) in emergency health professionals: a comprehensive methodological approach

**DOI:** 10.1186/s12913-025-13034-8

**Published:** 2025-07-01

**Authors:** Hamidreza Shabanikiya, Fatemeh Kokabisaghi, Fatemeh Dorri Khalilzadeh, Nazanin Khorasani, Jamshid Jamali

**Affiliations:** 1 Social Determinants of Health Research Center, Mashhad University of Medical Sciences, Mashhad, Iran; 2 Department of Health Economics and Management Sciences, School of Health, Mashhad University of Medical Sciences, Mashhad, Iran; 3 Student Research Committee, Mashhad University of Medical Sciences, Mashhad, Iran; 4 Department of Biostatistics, School of Health, Mashhad University of Medical Sciences, Mashhad, Iran

**Keywords:** Surveys and questionnaires, Translations, Reproducibility of results, Emergency medical services, Abbreviated Moral Injury Outcome Scale

## Abstract

**Background:**

Emergency health professionals face health risks and ethical dilemmas that can impact their well-being. This study evaluates the psychometric properties of the Persian version of the Abbreviated Moral Injury Outcome Scale (AMIOS) for these professionals.

**Methods:**

This scale development study was carried out in Mashhad, Iran, in 2024. The original AMIOS was translated using a forward-backward method. Content validity was assessed using the Content Validity Ratio (CVR) and Content Validity Index (CVI), based on assessments from 11 experts. Face validity was evaluated both quantitatively through the impact score and qualitatively. Construct validity was analyzed with Confirmatory Factor Analysis involving 280 participants. Reliability was measured by Cronbach’s alpha and McDonald’s omega coefficients for internal consistency and Intraclass Correlation Coefficient (ICC) for stability, based on data from 30 emergency health professionals.

**Results:**

The translated questionnaire demonstrated confirmed face validity. The content validity indices were acceptable with a CVR of 0.802 and a CVI of 0.762. Construct validity was supported, with item factor loadings exceeding 0.4. The internal consistency was strong: Cronbach’s alpha was 0.837 for Shame-related Outcomes, 0.788 for Trust Violation Outcomes, and an overall alpha of 0.881. The ICC was 0.859, indicating good reproducibility.

**Conclusions:**

The Persian version of AMIOS is a valid and reliable instrument for assessing the impact of moral injury in emergency healthcare settings. This tool facilitates a more comprehensive understanding and diagnosis of moral injuries.

**Supplementary Information:**

The online version contains supplementary material available at 10.1186/s12913-025-13034-8.

## Introduction

In recent years, the number of natural and man-made disasters has been increasing worldwide, resulting in significant financial, human, and casualty losses [1]. In almost all disasters, emergency services such as the police, fire brigade, and emergency medical services (including pre-hospital and hospital emergency services) are at the core of the disaster response system [2, 3]. Within these organizations, staff play a central role and are responsible for providing services to victims of accidents and disasters [4].

In general, working as a disaster and emergency response service provider involves operating in hazardous conditions with a wide range of health risks, both physical and mental [5, 6]. Additionally, ethical and moral challenges further complicate the difficulties of working in disaster response roles [7, 8]. study conducted by Karadag and Hakan underscores the ethical challenges associated with disasters. The authors argue that disasters vary considerably in their temporal, spatial, and magnitude-related dimensions, which complicates the ethical dilemmas they generate. Consequently, these dilemmas often defy simplistic or universal solutions, as the unique characteristics of each disaster necessitate context-specific approaches [9]. Emergency workers frequently encounter various ethical dilemmas, such as the choice between saving the life of an injured person and safeguarding their own health. During a natural disaster, such as an earthquake, medical personnel face overwhelming demands due to the surge in the number of injured individuals. Critical resources, including medical supplies, healthcare staff, and treatment facilities, become severely limited. In such circumstances, healthcare providers are confronted with the complex ethical challenge of resource allocation. They must make difficult decisions regarding prioritization, raising the critical question: Which patients should receive treatment first? This dilemma underscores the need for ethical frameworks and triage protocols to guide decision-making in high-pressure, resource-constrained environments. Additionally, they must make swift decisions to deliver the best possible care within constrained timeframes. Moreover, emergency workers aim to uphold their own values, the values of the injured individuals, and the values of their organizations while providing care. These are just a few examples of the numerous ethical challenges they may face in the field [3, 10]. Such challenges can lead to moral injury, making individuals susceptible to various mental health problems, including occupational stress, depression, Post-Traumatic Stress Disorder, and even suicidal behaviors [11, 12]. Moral injury is characterized as a profound psychological and emotional response that may emerge following exposure to events that fundamentally conflict with an individual’s deeply held moral or ethical principles. This condition often involves feelings of guilt, shame, or existential distress, stemming from the perception of having transgressed one’s own values or witnessing actions that violate one’s sense of right and wrong. Such experiences can have significant long-term impacts on mental health and well-being [13]. From an organizational perspective, moral challenges can result in decreased job satisfaction, increased job burnout, and ultimately a decline in employees’ performance and ability to provide effective emergency services [14]. The first step in mitigating the negative effects of ethical challenges on the health and efficiency of emergency health professionals is to understand the extent and severity of the consequences of facing ethical dilemmas [15]. To achieve this, it is essential to use valid tools to assess the adverse outcomes of moral challenges experienced by emergency health professionals.

Several studies have been conducted on moral injury among emergency and safety service workers. One such study aimed to determine the prevalence of moral injury among healthcare professionals and identify its predictive factors [16]. This survey was conducted on 595 healthcare professionals in the United States during the COVID-19 pandemic. Survey items included the Moral Injury Symptoms Scale–Health Professional (MISS-HP), items adapted from the Rushton Moral Resilience Scale (RMRS), and the Ethical Concerns Index (ECI). The study found that the prevalence of moral injury among healthcare professionals was 32.4%, with nurses experiencing the highest rates. Moral injury among healthcare workers was significantly associated with factors such as moral resilience, ECI scores, religious beliefs, and more than 20 years of work experience [16].

Another study conducted in Canada investigated the risk factors of moral injury among soldiers who participated in the Afghanistan war, using the Moral Injury Events Scale [17]. The study identified rank, experiences of sexual trauma and child maltreatment, feeling responsible for the death of a fellow soldier, and the inability to act appropriately in armed conflicts as key indicators of moral injury [17].

There is a limited availability of tools designed to measure moral injury, with the Abbreviated Moral Injury Outcome Scale (AMIOS) emerging as one of the most reliable instruments for this purpose [18]. Developed through a comprehensive international study involving experts from five countries, the AMIOS has demonstrated its applicability across diverse populations, including military personnel from four nations. However, its utilization among emergency health professionals in Iran remains notably absent. While a prior study by Akbari et al. in Iran focused on the psychometric assessment of moral injury symptoms, no research has yet explored the psychometric evaluation of moral injury outcomes within the Iranian context. It is critical to emphasize that symptoms and outcomes represent distinct dimensions of moral injury. The present study seeks to adapt the AMIOS for use among emergency health professionals in Iran, with the hypothesis that the scale will prove valid and reliable in this population.

The general structure of the Moral Injury Outcome Scale (MIOS) comprises three primary components: (1) PTSD Screen, (2) Core MIOS items, and (3) Functional Outcomes. Parts 1 and 3 are considered optional, while the full version of the scale includes all three sections. The clinical version consists of parts 1 and 2, and the abbreviated version (AMIOS) focuses solely on part 2, the core items. It is anticipated that the exclusion of the optional sections in the abbreviated version will not significantly affect the outcomes of psychometric testing, as the core items retain the essential elements necessary for assessing moral injury. This study aims to validate the AMIOS in Iran, addressing a critical gap in the assessment of moral injury outcomes among emergency health professionals.

Iran was chosen for this study because, according to the Institute for Environment and Human Security, Iran is categorized as a very high-risk country in terms of disasters [19]. Between 1990 and 2015, Iran experienced 208 natural disasters, resulting in 156,332 deaths and 4,464,390 injuries, giving it the highest natural disaster mortalityand morbidity rate in the Middle East during this period [20]. Applying the MIOS for healthcare workers in this disaster-prone country could enhance their ability to respond effectively to emergencies.

## Methods

The present study focused on the translation and validation of the scale from July to September 2024, targeting pre-hospital and hospital emergency health professionals in Iran. The AMIOS is designed to measure the impact of moral injury experienced by individuals in the past month. The original version of the AMIOS comprises 14 items, which are classified into two dimensions: shame-related outcomes and trust violation-related outcomes [18]. Dimension 1: Shame-related outcomes include items 1, 3, 7, 8, 12, 13, and 14, with a scoring range of 0–28. Dimension 2: Trust violation-related outcomes includes items 2, 4, 5, 6, 9, 10, and 11, also with a scoring range of 0–28 [18]. Each item on the scale is rated using a Likert scale (e.g., from 0 to 4), allowing respondents to indicate their level of agreement with the statements presented. Higher scores reflect greater levels of moral injury experienced. The overall score is calculated by summing the responses to all items. Currently, there are no proposed scoring categories or cutoffs [18]. The primary questionnaire used in this study is included in the article’s appendix. In this study, we employed the Forward-Backward translation technique to ensure the linguistic and conceptual accuracy of the AMIOS for Emergency Health Professionals when adapting it for use within Persian-speaking populations in the context of emergency health [21]. In studies focused on instrument development, sample sizes are determined separately for each stage, guided by established guidelines rather than fixed statistical formulas [22]. For content validity, a minimum of five experts is recommended, with ten being the preferred target [22]. Face validity is typically assessed using a sample of ten individuals from the target population [22]. Construct validity requires a range of ten to twenty participants per item [22], while repeatability and internal consistency are evaluated with a sample size of at least thirty participants from the target population [22].

In this study, the validity of the questionnaire was evaluated through multiple methods. Face validity was evaluated using both quantitative and qualitative approaches. Quantitative face validity was assessed by calculating the impact score, which measures the importance of each item in the questionnaire. Qualitative face validity was determined based on feedback provided by ten emergency health workers, who reviewed the items for clarity, relevance, and appropriateness. This dual approach ensured that the questionnaire was both statistically robust and practically meaningful for the target population. Quantitative content validity was determined using the Content Validity Ratio (CVR) and Content Validity Index (CVI), as evaluated by 11 experts in medical ethics, emergency medicine, and mental health [23, 24]. Construct validity was examined using Confirmatory Factor Analysis (CFA) with a sample of 280 participants from the target population. Cutoff points in alignment with the criteria set forth by Schermelleh-Engel et al. were employed for assessing goodness-of-fit indices in CFA [25]. Data for construct validity were collected through a combination of cluster sampling with proportional allocation and convenience sampling. The population was divided into clusters, with each hospital and its affiliated pre-hospital emergency health facilities (Emergency Medical Service, EMS) under Mashhad University of Medical Sciences serving as a cluster. Five hospitals and their corresponding EMS facilities were randomly selected, and the sample size for each was determined based on the number of staff in their emergency departments and EMS facilities to ensure proportional allocation. Within each cluster, a convenience sample of participants was selected based on staff availability and willingness to participate, ensuring representativeness across facilities.

For face validity, content validity, repeatability, and internal consistency, purpose-based sampling was employed. Participants were selected based on their expertise, relevance to the study objectives, or ability to provide meaningful insights. Experts in emergency medicine and healthcare research were chosen for face and content validity assessments, while participants from the target population were selected for repeatability and internal consistency testing to ensure the tool’s practicality and reliability. This approach ensured that participants were well-suited to provide accurate and relevant feedback for each evaluation phase, enhancing the study’s validity.

The reliability of the tool was assessed through internal consistency using Cronbach’s alpha and McDonald’s omega coefficients, while reproducibility was measured using the Intraclass Correlation Coefficient (ICC) based on responses from 30 pre-hospital and hospital emergency health professionals. Mashhad, the second-largest city in Iran, is located in the northeast and has a population exceeding three million, providing a diverse and representative setting for the study. All questionnaires were completed anonymously and confidentially, following the principles outlined in the Declaration of Helsinki [26]. The study protocol was approved by the Regional Ethics Committee of Mashhad University of Medical Sciences, Mashhad, Iran (approval number: IR.MUMS.FHMPM.REC.1402.122). Data was analyzed using SPSS version 25 and AMOS version 24 software, with a significance level of 0.05.

## Results

The translation of the AMIOS questionnaire involved a rigorous process, beginning with independent translations from English to Persian by two fluent English-speaking Persian translators. Following the initial translation, the questionnaire was re-translated into English by two other independent translators. The variants of these processes were then synthesized, and the median version underwent a review by a panel of experts for final approval. The completed Persian version of the questionnaire is presented in Table 1.

From the perspectives of the studied pre-hospital and hospital emergency health workers, all 14 items of the AMIOS questionnaire were rated as important, achieving an average impact factor of 3.5. This score exceeds the minimum acceptable threshold of 1.5 [27], thereby confirming the face validity of the questionnaire (Table 2).

**Table 1:**
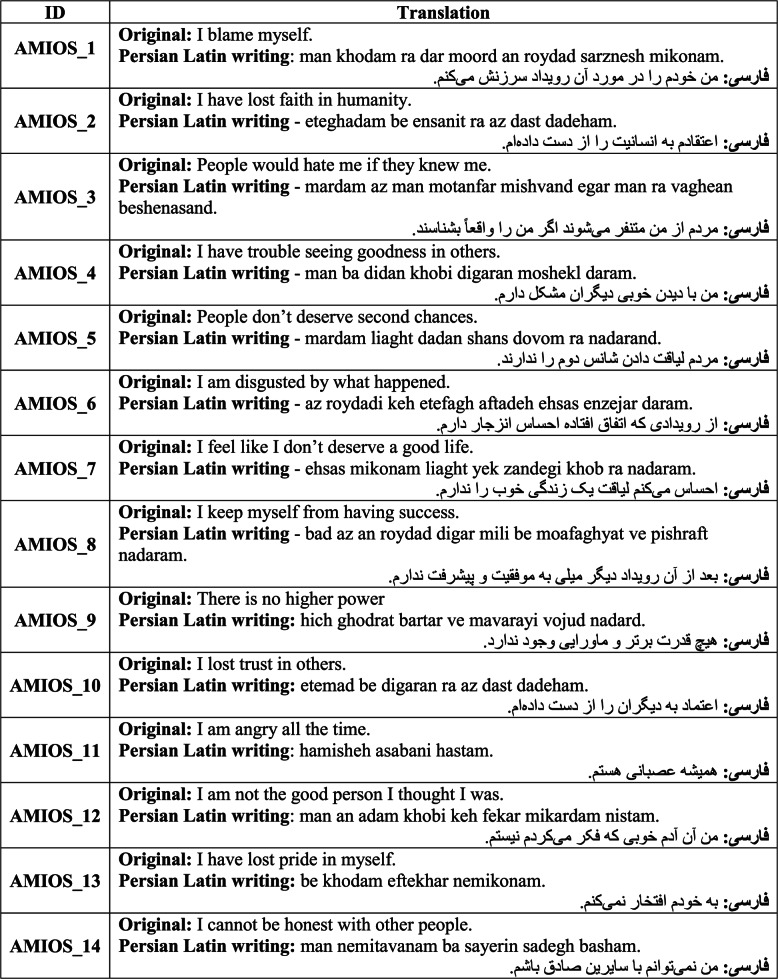
Translation of the Persian Version of AMIOS

For all items of the AMIOS questionnaire, Content Validity indices composed of the CVR and CVI fell within acceptable ranges. Specifically, all CVR values surpassed 0.59, following Lavashe’s criteria [23], while CVI values exceeded 0.79 [24]. The average CVR was calculated at 0.802, and the average CVI at 0.762, based on the evaluations of 11 experts in medical ethics, emergency medicine, and mental health (see Table 2).

**Fig. 1:**
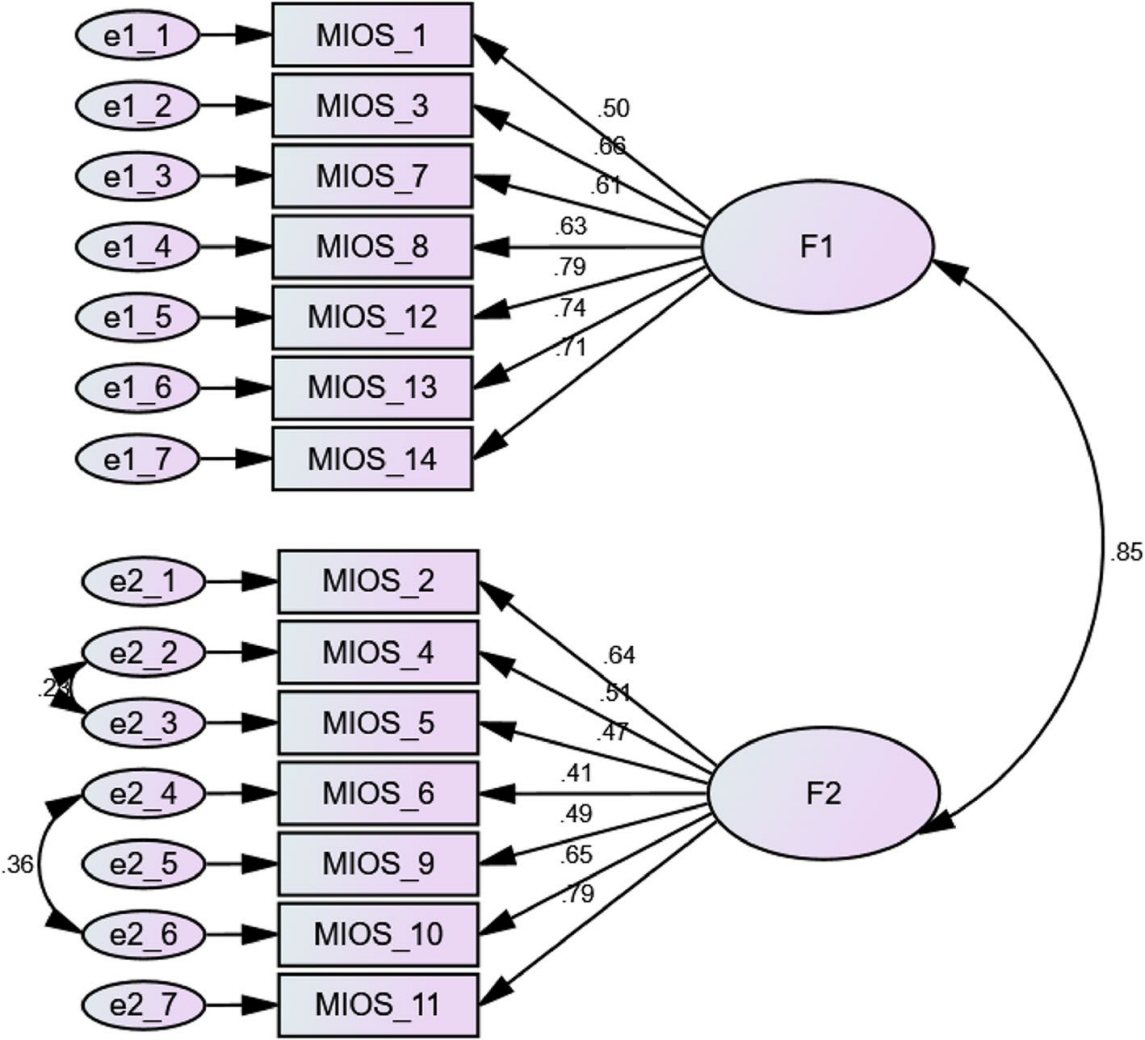
Conceptual model in construct validity assessment using CFA

The assessment of construct validity involved 280 participants, comprising 40% men (*n* = 112), and 30.4% single (*n* = 85). The mean age of participants was 33.10 ± 7.29 years. For further demographic details see Table 3. All item factor loading values exceeded 0.4, which confirms the construct validity of the instrument (see Fig. 1; Table 2).

**Table 2:** Validity evaluation of the AMIOS indicators

F	Item	Impact Score	CVR	CVI (Relevancy)	Loading Factor	Result
F1 Shame-related Outcomes	AMIOS_1	3.36	0.818	0.909	0.495	Confirmed
	AMIOS_3	2.34	0.636	0.818	0.655	Confirmed
	AMIOS_7	3.36	1.000	1.000	0.605	Confirmed
	AMIOS_8	2.80	0.818	0.909	0.627	Confirmed
	AMIOS_12	3.96	1.000	1.000	0.794	Confirmed
	AMIOS_13	4.50	1.000	1.000	0.74	Confirmed
	AMIOS_14	3.28	1.000	0.909	0.713	Confirmed
F2 Trust Violation-related Outcomes	AMIOS_2	3.36	1.000	1.000	0.641	Confirmed
	AMIOS_4	3.44	1.000	1.000	0.507	Confirmed
	AMIOS_5	3.28	0.636	0.909	0.473	Confirmed
	AMIOS_6	3.36	0.818	0.818	0.415	Confirmed
	AMIOS_9	3.28	1.000	1.000	0.488	Confirmed
	AMIOS_10	4.50	1.000	1.000	0.645	Confirmed
	AMIOS_11	3.36	1.000	1.000	0.795	Confirmed

The goodness-of-fit indices from the confirmatory factor analysis model are summarized in Table 4. The internal consistency was strong, with Cronbach’s alpha and McDonald’s omega coefficients of 0.837 and 0.839 for Shame-related Outcomes, 0.788 and 0.798 for Trust Violation Outcomes, and an overall alpha of 0.881 for both coefficients. All values were higher than the acceptable threshold of 0.7 [28]. Finally, the ICC for the tool, which assesses reproducibility, was at 0.859, demonstrating satisfactory reproducibility of the instrument [29].

**Table 3:** Demographic information of participants in the construct validity assessment

Variable	Category	Number	Percent
Gender	Male	112	40
	Female	168	60
Marriage	Single	85	30.4
	Married	195	69.6
Job	Physician	12	4.3
	Nurse	229	81.8
	Paramedics	39	13.9
Education	Associate	23	8.2
	Bachelor	217	77.5
	Master	27	9.6
	PhD	1	0.4
	Post do	12	4.3

**Table 4:** Goodness indices of model fit in CFA to assess construct validity AMIOS

Index	Estimation	Acceptable Value	Result
$$\:\raisebox{1ex}{$\varvec{\upchi\:}2$}\!\left/\:\!\raisebox{-1ex}{$\mathbf{d}\mathbf{f}$}\right.$$	3.031	Between 1 and 5	acceptable
CFI	0.882	Greater than 0.8	acceptable
TLI	0.855	Greater than 0.8	acceptable
RMSEA	0.091	Smaller than 0.10	acceptable

## Discussion

In this study, the psychometric properties of the Iranian version of the AMIOS for emergency health professionals were evaluated. The face validity of the translated questionnaire CVR, CVI, construct validity, reliability, and ICC were all confirmed. In the following section, we present a comparative analysis of the current tool with five similar instruments: ECI, MISS-HP, EMIS-M, and MISS-HP-Chinese version. This comparison highlights the distinct features, strengths, and limitations of each tool, providing a comprehensive understanding of their applicability in assessing moral injury across different contexts and populations. By examining these instruments side by side, we aim to underscore the unique contributions of the current tool and its potential to address gaps in the measurement of moral injury, particularly within emergency healthcare settings. One of the studies that developed a tool (ECI) for assessing the negative impacts of ethical challenges in healthcare settings was conducted by Rushton et al. [16]. Their study aimed to explore the frequency of moral injury among healthcare workers, examine the connections between predictive factors of moral injury, and assess the potential protective role of moral resilience against moral injury. The study was conducted during the COVID-19 pandemic and included items on outcomes, the MISS-HP, and predictors such as demographics, items from the RMRS, and the ECI. Among the tools used in this study, the ECI was the only one specifically developed and presented by Rushton. The ECI was created by three experts in medicine, bioethics, and nursing, along with a literature review. However, in developing the ECI, only construct validity was assessed. Other types of validity, such as content validity and face validity, as well as reliability, were not evaluated [16]. In contrast, the tool in the present study was assessed for both content validity and reliability (internal consistency and temporal stability).

Another tool, the MISS-HP, was developed by Mantri et al. [30] to evaluate the psychometric properties of an assessment tool for moral injury symptoms among healthcare professionals. The MISS-HP is an adapted version of the Moral Injury Symptom Scale–Military–Short Form (MISS-M-SF) for healthcare professionals [31]. The language of the MISS-M-SF was adjusted to specifically suit healthcare professionals. Construct validity and internal consistency were investigated in their study. The internal consistency, measured by Cronbach’s alpha, was 0.75, which is above the minimum acceptable threshold of 0.70. Through Exploratory Factor Analysis (EFA), three factors were identified and confirmed by confirmatory factor analysis. However, content and face validity were not assessed in their study, which is a limitation compared to the present study. On the other hand, their study demonstrated convergent and discriminant validity, which is a strength. Convergent validity was established through strong correlations between the MISS-HP and clinician burnout and the Expressions of Moral Injury Scale. Weak correlations between the MISS-HP and religiosity, depressive symptoms, and anxiety symptoms indicated discriminant validity.

Another validated tool is the Expressions of Moral Injury Scale–Military (EMIS-M) [32]. This tool was designed to evaluate moral injury symptoms among military personnel. The initial version of the EMIS-M was developed in a four-phase process based on input from subject matter experts. To assess content validity, 33 subject matter experts with a strong understanding of moral injury were selected from 300 invited experts. While this appears to be a strength of the study, a limitation is the lack of quantitative content validity assessment methods, such as the CVR or CVI, to achieve consensus among experts. In this regard, the present study is stronger. Additionally, face validity was not evaluated in Currier et al.‘s study, unlike in our study. Both EFA and parallel analysis were used to investigate the underlying factors of the EMIS-M, ultimately identifying two distinct factors. The bifactor model of the EMIS-M was validated through CFA. The EMIS-M demonstrated strong internal consistency, with Cronbach’s alpha values of 0.98, indicating excellent reliability. To assess temporal stability, the target population completed the questionnaire at two time points, six months apart. A total of 183 individuals participated in the test-retest assessment, and the results showed strong correlations between the scores of the two subscales and the total score at both time points. In both the present study and Currier et al.‘s study, reliability (internal consistency and temporal stability) and construct validity were assessed and confirmed. A strength of Currier et al.‘s study was the assessment of convergent and discriminant validity, which was not conducted in the present study. This omission is justified, as our study focused on translating and adapting a pre-existing tool rather than developing a new one.

The MISS-HP-Chinese Version was developed in a study aimed at assessing moral injury symptoms among healthcare professionals in China [33]. The World Health Organization’s (WHO) four-step process [34] was followed to translate the English version into Chinese. An online survey targeting physicians and nurses was conducted during the COVID-19 pandemic using the translated version. Reliability was evaluated through internal consistency and test-retest methods. The Cronbach’s alpha for the scale was deemed acceptable (0.71), as were the ICCs for test-retest reliability of the overall scale. Similarly, in the present study, the WHO’s four-step process for tool translation was followed. Internal reliability was assessed and confirmed using Cronbach’s alpha, while external reliability was evaluated through ICC calculation using the test-retest technique. However, in Zhizhong et al.‘s study, content and face validity were not assessed, unlike in our study. This difference may be due to the fact that their study did not aim to adapt the MISS-HP for a new group of individuals, whereas our study focused specifically on hospital and pre-hospital emergency health professionals. EFA and CFA were conducted to assess structural validity in Zhizhong et al.‘s study. EFA revealed three factors, while CFA demonstrated a good fit for the data. Similarly, in our study, CFA was conducted to evaluate the two factors from the original version of the MIOS, and the results validated the two-factor structure.

Overall, among the strengths of the tool adopted in the present study, compared to some of the aforementioned studies, is the inclusion of content and face validity analysis, which were not considered in most of the other studies. Additionally, many of the studies examining moral injury among healthcare professionals were conducted during the COVID-19 pandemic [35, 36]. The unique conditions during pandemics and crises may limit the generalizability of their findings to normal conditions. In contrast, our study was conducted under typical working conditions, enhancing its applicability to routine settings.

While this study provides valuable insights into the translation and validation of the AMIOS for Persian-speaking emergency health professionals, several limitations should be acknowledged. First, the use of convenience sampling within clusters may introduce selection bias, as participants were not randomly selected at the individual level. Second, the study was conducted in a single geographic region (Mashhad), which may limit the generalizability of the findings to other regions or cultural contexts. Third, the reliance on self-reported data may be subject to response bias, particularly given the sensitive nature of moral injury. Fourth, this study did not address the measurement invariance of the questionnaire, which is one of the sub-aspects of validity. Finally, the cross-sectional design of the study limits the ability to assess the long-term stability of the scale. Future studies should consider longitudinal designs and include diverse populations to further validate the AMIOS and address these limitations.

## Conclusion

The Iranian version of AMIOS has been established as a reliable and valid tool for assessing the impact of moral injury in emergency healthcare settings. This scale aids healthcare professionals in identifying high levels of moral injury that may hinder social or occupational functioning, thus necessitating clinical intervention. Emergency health managers can utilize the AMIOS to conduct practical studies that identify personnel experiencing moral injury. This capability allows for the development of evidence-based strategies aimed at mitigating the risks associated with moral injury among their staff. Moreover, given that emergency health professionals frequently encounter ethical challenges and health risks in their work, which adversely affect their psychological well-being, the AMIOS serves as a crucial resource in addressing these concerns.

This study confirms the psychometric validation of the Persian version of the AMIOS, demonstrating its acceptability and reliability for assessing moral injury among emergency healthcare providers in Iran. By implementing the validated AMIOS, healthcare organizations can effectively identify moral injury within their settings, enabling them to introduce targeted interventions and support systems that improve the mental health and overall well-being of healthcare professionals. Further research is essential to explore the psychometric properties of the AMIOS across diverse settings and conditions, ensuring its robust application in various contexts.

## Supplementary Information

Supplementary Material 1

## Data Availability

The dataset analyzed in this article is available from the corresponding author upon reasonable request, contingent upon obtaining permission from the Mashhad Regional Committee for Medical Research Ethics.

## Abbreviations

MISS-HP: Moral Injury Symptoms Scale–Health Professional
RMRS: Rushton Moral Resilience Scale
ECI: Ethical Concerns Index
AMIOS: Abbreviated Moral Injury Outcome Scale
CVR: Content Validity Ratio
CVI: Content Validity Index
CFA: Confirmatory Factor Analysis
ICC: Intraclass Correlation Coefficient
EMS: Emergency Medical Service
EMIS-M: Moral Injury Scale–Military
EFA: Exploratory Factor Analysis
