# Supplementary material for: Translation and validation for Persian version of Abbreviated Moral Injury Outcome Scale (AMIOS) in emergency health professionals: a comprehensive methodological approach
Source: BMC Health Serv Res. 2025 Jul 1;25:850. doi: 10.1186/s12913-025-13034-8 (PMC12217525; doi:10.1186/s12913-025-13034-8)
Supplement: Supplementary file 1 — Supplementary Material 1 [file 12913_2025_13034_MOESM1_ESM.pdf]

MIOS - F

This questionnaire asks about experiences you may have had after a very stressful experience in which:

- **You did something (or failed to do something)** that went against your moral code or values (e.g., you harmed someone or failed to protect someone from harm), or
- **You saw someone** (or people) do something or fail to do something that went against your moral code or values (e.g., you witnessed cruel behaviour), or
- **You were directly affected** by someone doing something or failing to do something that went against your moral code or values (e.g., being betrayed by someone you trusted).

---

1. Have you had an experience (or experiences) as described above?

❑ Yes ❑ No →

STOP
You do not have to complete this questionnaire.

↓

Please answer questions A-C while thinking about the worst event that currently bothers you the most. This could be one of the examples above, or some other very stressful experience that went against your core values.

A. Did the event involve something you did or failed to do?
❑ Yes ❑ No

B. Did the event involve observing someone else acting (or failing to act)?
❑ Yes ❑ No

C. Did the event involve being directly impacted by someone else (or people) acting (or failing to act)?
❑ Yes ❑ No

For events that had multiple features, which aspect was the worst (A, B, or C) ____?

2. In what year did this event happen ________?

3. Did the event involve actual or threatened death, serious injury, or sexual violence?
❑ Yes ❑ No

4. In the past month, have you...

	Yes	No
**A.** had nightmares about the event or thought about the event when you did not want to?	❑	❑
**B.** tried hard not to think about the event or went out of your way to avoid situations that reminded you of the event(s)?	❑	❑
**C.** been constantly on guard, watchful, or easily startled?	❑	❑
**D.** felt numb or detached from people, activities, or your surroundings?	❑	❑
**E.** felt guilty or unable to stop blaming yourself or others for the event(s) or any problems the event(s) may have caused?	❑	❑

5. If you feel comfortable, briefly describe the worst event:

Keeping this worst event in mind, please read each of these statements and circle one of the numbers to the right to indicate how much you would agree with the statement in the past month.

		Strongly Disagree	Disagree	Neither Agree or Disagree	Agree	Strongly Agree
1.	I blame myself.	0	1	2	3	4
2.	I have lost faith in humanity.	0	1	2	3	4
3.	People would hate me if they really knew me.	0	1	2	3	4
4.	I have trouble seeing goodness in others.	0	1	2	3	4
5.	People don't deserve second chances.	0	1	2	3	4
6.	I am disgusted by what happened.	0	1	2	3	4
7.	I feel like I don't deserve a good life.	0	1	2	3	4
8.	I keep myself from having success.	0	1	2	3	4
9.	There is no higher power.	0	1	2	3	4
10.	I lost trust in others.	0	1	2	3	4
11.	I am angry all the time.	0	1	2	3	4
12.	I am not the good person I thought I was.	0	1	2	3	4
13.	I have lost pride in myself.	0	1	2	3	4
14.	I cannot be honest with other people.	0	1	2	3	4

How much has this experience made it hard for you to function in each of the following areas? Circle one number for each item below. If an area is not applicable, circle N/A.

		Not at all			Somewhat			Extremely	
1.	Romantic relationships with spouse or partner	0	1	2	3	4	5	6	N/A
2.	Relationships with your children	0	1	2	3	4	5	6	N/A
3.	Relationships with other family members	0	1	2	3	4	5	6	N/A
4.	Friendships or socializing	0	1	2	3	4	5	6	N/A
5.	Professional (work, education, training)	0	1	2	3	4	5	6	N/A
6.	Hobbies and recreation activities that you do alone	0	1	2	3	4	5	6	N/A
7.	Hobbies and recreation activities that you do with others	0	1	2	3	4	5	6	N/A
8.	Religious faith/spirituality	0	1	2	3	4	5	6	N/A
9.	Day to day activities, such as chores, errands, finances, health	0	1	2	3	4	5	6	N/A

END OF ASSESSMENT

FOR ADMINISTRATORS

INSTRUCTIONS AND SCORING
The Moral Injury Outcome Scale (MIOS)

General Scale Structure

1. **Exposure Classification.** The first question is a dichotomous yes/no to determine whether an individual should complete the MIOS. **Persons who answer "no" should not complete the MIOS.** The next questions of Page 1 determine which type of moral injury experience(s) is most currently distressing. These initial questions on page 1 are not scored. The clinical and abbreviated versions of the MIOS do not contain questions related to the date and description of the event.
2. **PTSD Screen (optional).** The Primary Care PTSD Screen for DSM-5 (PC-PTSD-5; Prins et al., 2016)*. The clinical and abbreviated versions of the MIOS do not contain this section.
3. **Core MIOS items.** The core 14-item Likert scale of the MIOS (see below for scoring information).
4. **Functional Outcomes (optional).** The 9 Likert items following the MIOS are based on the Brief Inventory of Psychosocial Functioning (B-IPF; Kleiman et al., 2020), with items 5-9 adapted/added. The abbreviated version of the MIOS does not contain this section.

Scale Versions
Depending on administrators needs and contexts, different versions of the MIOS have been created to faciliate optimal data collection needs. In each of the versions, the core 14 items of the MIOS are always included.

	Full Version (F)	Clinical (C)	Abbreviated (A)
**Exposure Classification**	Extended classification of potentially morally injurious experience; **includes date and description of event**	Only classification of potentially morally injurious experience	Only whether a potentially morally injurious experience was experienced
**PTSD Screen**	Yes	No	No
**Core MIOS**	FULL 14 ITEMS OF MIOS ALWAYS INCLUDED		
**Functional Outcomes**	Yes	Yes	No

Scoring

MIOS Scoring Instructions:
The 14-item MIOS is comprised of two subscales, and scale scores are indexed by the sum of item scores as follows:
Subscale 1: Shame-related Outcomes – items 1, 3, 7, 8, 12, 13, 14 [range 0-28]
Subscale 2: Trust Violation-related Outcomes – items 2, 4, 5, 6, 9, 10, 11 [range 0-28]
Total Score: All items [range 0-56]
Higher scores indicate greater levels of current moral injury.
There are currently no proposed scoring categories or cutoffs.

Functional Outcomes Scoring Instructions:
The 9-item functional outcome measure is adapted from the Brief Inventory of Psychosocial Functioning (B-IPF). Responses on these items may be considered independently for clinical purposes. Some items may not be applicable for different persons. An overall impression of functional impairment can be obtained by calculating as follows:
1. Sum responses for all answered items.
2. Divide by the total number of items answered.
3. Multiply by 10. [range 0-60]

Higher scores indicate more functional impairment.
There are currently no proposed scoring categories or cut offs.

FOR ADMINISTRATORS

INSTRUCTIONS AND SCORING
The Moral Injury Outcome Scale (MIOS)

How to Cite this Scale

Litz BT, Plouffe RA, Nazarov A, Murphy D, Phelps A, Coady A, Houle SA, Dell L, Frankfurt S, Zerach G, Levi-Belz Y and The Moral Injury Outcome Scale Consortium. (2022). Defining and Assessing the Syndrome of Moral Injury: Initial Findings of the Moral Injury Outcome Scale Consortium. Front. Psychiatry 13:923928. doi: 10.3389/fpsyt.2022.923928.

Moral Injury Outcome Scale Consortium members:
Andrea Ashbaugh, Julie Yeterian, Danielle Berke, Jessica Carney, Mackenzie Cummings, Ruth Chartoff, Stephanie Ellickson-Larew, Breanna Grunthal, Maya Bina N. Vannini, Frank Weathers, Luke Rusowicz-Orazem, Patrick Smith, Fardous Hosseiny, Lisa King, J. Don Richardson, Alexandra McIntyre-Smith, David Forbes, Kim Jones, Ellie Lawrence-Wood, Kelsey Madden, Kim Murray.

Other References

*(included with permission)

Prins, A., Bovin, M. J., Smolenski, D. J., Marx, B. P., Kimerling, R., Jenkins-Guarnieri, M. A., Kaloupek, D. G., Schnurr, P. P., Kaiser, A. P., Leyva, Y. E., & Tiet, Q. Q. (2016). The Primary Care PTSD Screen for DSM-5 (PC-PTSD-5): Development and Evaluation Within a Veteran Primary Care Sample. Journal of general internal medicine, 31(10), 1206–1211. https://doi.org/10.1007/s11606-016-3703-5.

Kleiman, S. E., Bovin, M. J., Black, S. K., Rodriguez, P., Brown, L. G., Brown, M. E., Lunney, C. A., Weathers, F. W., Schnurr, P. P., Spira, J., Keane, T. M., & Marx, B. P. (2020). Psychometric properties of a brief measure of posttraumatic stress disorder-related impairment: The Brief Inventory of Psychosocial Functioning. Psychological services, 17(2), 187–194. https://doi.org/10.1037/ser0000306.
